# The efficacy of temperature-guided preventive care in reducing diabetic foot ulcer incidence and prolonging ulcer-free survival: a systematic review and meta-analysis of randomized controlled trials

**DOI:** 10.3389/fmed.2026.1830535

**Published:** 2026-06-22

**Authors:** Sheng Li, Dan Jiang, Yalan Liu, Rui Zuo

**Affiliations:** 1 Department of Burn and Plastic Surgery, West China Hospital of Sichuan University-Ziyang Hospital, Ziyang Central Hospital, Ziyang, Sichuan, China; 2 Clinical Medical College and Affiliated Hospital of North Sichuan Medical College, Nanchong, Sichuan, China

**Keywords:** diabetes mellitus, diabetic foot ulcers, meta-analysis, skin temperature monitoring, temperature-guided preventive care, ulcer recurrence

## Abstract

**Introduction:**

Recurrence of diabetic foot ulcers (DFU) is a major clinical challenge, often leading to amputation. Temperature-guided preventive care based on skin temperature monitoring has been proposed as an early warning tool that can trigger preventive actions before overt ulceration develops. We conducted a systematic review and meta-analysis to evaluate its efficacy in preventing DFU recurrence in high-risk individuals.

**Methods:**

We systematically searched PubMed, Embase, the Wiley Online Library, and Web of Science for randomized controlled trials comparing temperature-guided preventive care based on skin temperature monitoring with standard care. The primary outcome was time to first ulcer recurrence, with the hazard ratio (HR) as the effect measure. The secondary outcome was overall recurrence incidence, measured by the risk ratio (RR).

**Results:**

Six randomized controlled trials involving a total of 917 participants were included. The meta-analysis demonstrated a significant protective effect for temperature-guided preventive care. The intervention group had a significantly lower hazard of ulcer recurrence compared with the control group (HR 0.63; 95% CI: 0.49–0.82; *p* = 0.001). Similarly, the overall risk of recurrence was significantly reduced in the intervention group (RR 0.64; 95% CI: 0.52–0.80; *p* = 0.001).

**Conclusion:**

Temperature-guided preventive care appears to be an effective, patient-driven intervention that may reduce the risk and incidence of DFU recurrence in high-risk individuals. These findings support the potential integration of this simple, proactive tool into standard preventive care protocols to mitigate the severe consequences of this complication.

## 1 Introduction

Diabetes mellitus (DM) is one of the most common chronic metabolic diseases worldwide, and the incidence rate has been continuously rising in recent years. DM is one of the leading causes of death and disability worldwide, with an estimated 529 million people living with the condition in 2021 (1). Common complications of diabetes include diabetic cardiovascular complications, diabetic cerebrovascular disease, diabetic nephropathy, diabetic eye disease, diabetic foot, and diabetic neuropathy (2, 3). Diabetic foot ulcer (DFU) is one of the most serious and costly complications of diabetes, affecting approximately 18.6 million individuals globally each year. DFU profoundly diminishes patients' quality of life through chronic pain, reduced mobility, and an increased risk of depression and social isolation. Furthermore, DFUs impose an enormous economic burden on healthcare systems, driven by high costs associated with prolonged hospitalization, complex wound care, surgical interventions, and long-term rehabilitation. For instance, a study in the United States reported that the average in-hospital cost for a single DFU episode was approximately US$10,800 (4). The clinical progression of these ulcers is frequently unfavorable. Infection occurs in approximately 50% to 60% of cases, and approximately 20% of moderate-to-severe infections result in lower extremity amputation (5). Importantly, beyond the risk of infection and amputation, patients with DFU also face a poor long-term prognosis, with evidence suggesting a 5-year mortality rate of approximately 30%, comparable to that reported for cancer in general (6).

While the initial healing of a DFU is a clinical success, the paramount challenge lies in its high rate of recurrence. Up to 70% of patients experience a subsequent ulcer within 5 years (7), a stark figure that reframes ulcer healing not as a cure, but as a temporary “remission period” after which the patient enters a state of continuous high risk. This high susceptibility to recurrence is rooted in the underlying, often irreversible, pathophysiology of the diabetic foot, primarily diabetic peripheral neuropathy, which impairs protective sensation, and peripheral artery disease, which compromises tissue perfusion and healing. Pathophysiologically, DFU results from a combination of diabetic peripheral neuropathy, repetitive mechanical stress, and altered foot biomechanics and is characterized by difficult-to-heal lesions, high recurrence rates, and poor prognosis (8). DFUs are also one of the most common precursors to diabetes-related amputations (9). Consequently, the healed foot remains vulnerable to repetitive mechanical stress that goes unnoticed by the patient. In addition, neuropathy-related autonomic dysfunction may impair sweating and contribute to dry, fragile skin, thereby further weakening the skin barrier and increasing susceptibility to tissue injury. The majority of non-traumatic DFUs are preceded by localized inflammation and enzymatic autolysis of tissue, which generate a quantifiable increase in local skin temperature before overt skin breakdown occurs (10, 11). This thermal warning is particularly crucial because other cardinal signs of inflammation, such as pain, are often masked or unreliable in individuals with diabetic neuropathy (12). Although preventive strategies such as proper foot care, glycemic control, and early risk factor management remain fundamental, their effectiveness in preventing recurrence is often insufficient, underscoring the need for more proactive and patient-driven interventions.

These early neuropathic and inflammatory changes may provide a window for earlier detection and preventive intervention before overt ulceration develops. In particular, early small-fiber and autonomic dysfunction may already compromise sweating, skin integrity, and protective sensation, thereby increasing susceptibility to unnoticed tissue injury and reinforcing the rationale for temperature-based monitoring approaches in high-risk patients (13, 14).

Accordingly, monitoring local skin temperature has been proposed as a practical approach for identifying patients at imminent risk of ulceration (10). In clinical practice, the early signs of ulcer development are often difficult to detect during routine podiatric assessment, whereas temperature measurement may provide an objective and quantifiable early warning signal (15–17). By self-monitoring foot skin temperatures at home, patients may detect abnormal temperature elevations and take timely preventive actions, such as reducing ambulatory activity or implementing pressure-relief strategies, before a wound develops (16). Notably, three randomized controlled trials (RCTs) have demonstrated that temperature-guided preventive care significantly reduces the incidence of foot ulcer recurrence in people with diabetes (16, 18, 19).

In terms of practical implementation, temperature-guided preventive care represents an accessible strategy for detecting early signs of impending ulceration and helping to prevent the occurrence or recurrence of DFU (16). Broadly, current home temperature-monitoring approaches can be categorized into two modes: static measurement and dynamic measurement. Static measurement is performed while the patient is at rest, with temperature assessed at a single time point, typically using handheld infrared thermometers (20). In contrast, dynamic measurement aims to support earlier detection and timely intervention through continuous or repeated monitoring during daily activity and includes technologies such as direct-contact sensors, smart mats, and wearable devices such as smart insoles or socks (21–23). These systems can capture temperature changes across multiple sites of the foot and, in some cases, enable remote data transmission and automated warning signals, making them increasingly suitable for routine self-monitoring in daily life (24).

While several studies have individually demonstrated the effectiveness of temperature-guided preventive care based on skin temperature monitoring, the overall magnitude of the effect and the consistency of these findings across different studies remain to be systematically synthesized. A comprehensive systematic review and meta-analysis are therefore needed to provide a more robust and precise estimate of the treatment effect. Therefore, this article aimed to systematically review the existing literature and conduct a meta-analysis to quantify the efficacy of temperature-guided preventive care based on skin temperature monitoring in reducing recurrent diabetic foot ulcers and prolonging ulcer-free survival in high-risk individuals. The findings are intended to provide a solid evidence base for its clinical application and to inform future guideline recommendations.

## 2 Methods

This systematic review and meta-analysis was conducted according to the Preferred Reporting Items for Systematic Reviews and Meta-Analyses (PRISMA) 2020 Statement (25). The study protocol was registered in the International PROSPERO, registration number: CRD420251154072. In this study, two authors independently searched databases, selected studies, extracted data, and assessed the risk of bias, while the third author resolved any disagreements that arose during the process.

### 2.1 Ethical statement

Since our meta-analysis was based on data from previously published studies, ethical approval and informed consent were not required for this study.

### 2.2 Data sources and search strategy

A systematic literature search was conducted in four electronic databases: PubMed, Embase, the Wiley Online Library, and Web of Science. The search covered all records from database inception through August 30, 2025, with no language restriction. The full search strategies for each database are detailed in Supplementary File 1. The reports were not limited to any language, and there were no publication date restrictions. Additionally, we performed a manual search of reference lists from key articles to identify other relevant studies.

### 2.3 Study selection criteria

Initially, the titles and abstracts of all articles retrieved from these databases were screened, and subsequently, a second screening was conducted for the full text. Literature selection was based on the following inclusion and exclusion criteria.

Inclusion criteria:

- Studies that enrolled patients with type 1 or type 2 diabetes mellitus at risk of developing a diabetic foot ulcer;
- Studies that reported the incidence of diabetic foot ulcer recurrence as an outcome;
- Studies that evaluated the efficacy of temperature-guided preventive care based on skin temperature monitoring;
- Study design was RCTs.

Exclusion criteria:

- Reviews, meta-analyses, case reports, letters, comments, basic-research articles, and conference abstracts;
- Studies with insufficient data for analysis;
- Studies reporting duplicate patient populations.

The primary outcome was the time to the first foot ulcer recurrence. The effect measure was the HR with its corresponding 95% CI. Secondary outcomes were the overall incidence of foot ulcer recurrence at the end of each study's follow-up period. The effect measure was the RR with its corresponding 95% CI, calculated from the number of events and total participants in each group.

### 2.4 Data extraction and quality assessment

Using a predefined data extraction form, two independent reviewers extracted data on the following variables: (1) Study characteristics (authors; country in which the study was performed; study design; follow-up time; sample size). (2) Patient characteristics (patient population; age; sex; proportion of patients with type 2 DM; neuropathy rates; peripheral artery disease rates). In addition, intervention-level data were extracted, including the monitoring device, measurement frequency, anatomical measurement sites, threshold definition for abnormal temperature, preventive actions triggered by abnormal findings, and details of standard care or control care.

The quality of the included studies was assessed using the Cochrane risk of bias tool. Two authors independently evaluated each study across seven domains: random sequence generation, allocation concealment, blinding of participants and personnel, blinding of outcome assessment, incomplete outcome data, selective reporting, and other potential sources of bias. Each domain was categorized as having a low, high, or unclear risk of bias. Discrepancies were resolved by consensus or by involving a third author.

### 2.5 Statistical analysis

For the primary outcome, the HR with 95% CIs for the time to first ulcer recurrence were pooled. For secondary outcomes, the RRs with 95% CIs were calculated from the number of ulcer recurrence events and the total number of participants in each group. HRs and RRs were extracted directly from the publications where available. For studies that only provided Kaplan-Meier curves, HRs were estimated using established statistical methods (26). The degree of heterogeneity was assessed using the I^2^ test, with values < 30%, 30%−50%, and >50% indicating low, moderate, and high levels of heterogeneity, respectively. If I2 < 50%, the fixed-effect model was used; otherwise, the random-effect model was applied. We performed a sensitivity analysis to assess the robustness of our analysis, and we employed Begg's test and funnel plots to examine publication bias. Due to the limited number of included studies, further subgroup analyses were not performed.

The RevMan 5.3 (The Cochrane Collaboration, Oxford, UK) and Stata 18 (StataCorp, College Station, TX, USA) software were used to conduct all statistical analyses. *P* < 0.05 was considered statistically significant.

### 2.6 Patient and public involvement

It was not appropriate or possible to involve patients or the public in the design, conduct, or reporting, or dissemination plans of this study.

## 3 Results

### 3.1 Study selection and characteristics

**Figure 1:**
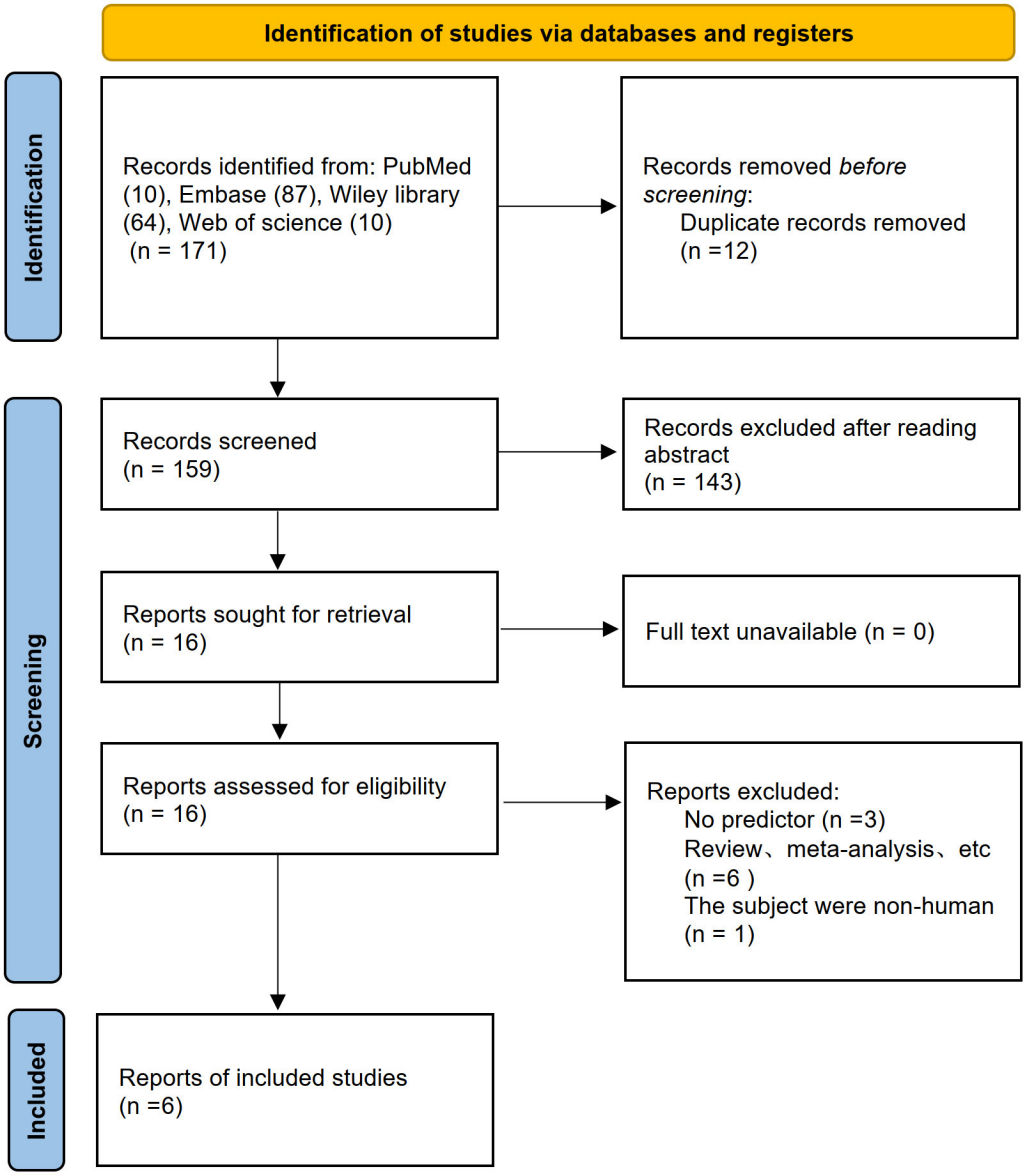
PRISMA flowchart of the study selection process.

The study selection process is shown on the PRISMA flow chart (Figure 1). A total of 171 articles were initially identified; 12 articles were removed due to duplicates. Following title and abstract screening based on predefined inclusion and exclusion criteria, 16 articles remained. We conducted a full-text assessment of the remaining 16 studies, and 10 were excluded. Finally, 6 studies involving a total of 917 patients were considered eligible and included in the meta-analysis (16, 18, 27–30). The baseline characteristics of the included studies are summarized in Table 1. All included articles were RCTs published between 2007 and 2023. The mean age of participants varied across studies, ranging from 58.25 to 68.45 years, and the male populations ranged from 48.3% to 80.5%. The follow-up periods ranged from 24 to 78 weeks. The majority of participants were diagnosed with Type 2 diabetes, with the proportion ranging from 70% to 100%. The prevalence of key risk factors varied, notably peripheral artery disease and neuropathy, which were reported in 0% to 31.5% and 33.3% to 100% of participants across studies. The intervention protocols varied across studies in terms of monitoring device, measurement frequency, measurement sites, threshold definition, and actions triggered by abnormal temperature findings. In most trials, temperature monitoring was embedded within a broader preventive care protocol rather than used as an isolated measurement procedure. Details of the temperature monitoring and intervention protocols are summarized in Supplementary Table 1.

**Table 1:** Characteristics of included studies.

References	Country	Study design	Sample size (Experiment/ Control)	Follow-up (weeks)	Male/ Female	Age, years (mean)	BMI	Smoking status(%)	T2DM (%)	PAD (%)	Neuropathy (%)	DOI
Armstrong et al. (18)	USA	RCT	225(112/113)	60	207/18	68.45	NR	NR	100	16.4	100	10.1016/j.amjmed.2007.06.028
Lavery et al. (16)	USA	RCT	117(59/58)	60	72/45	65.2	NR	NR	95	5.1	100	10.2337/dc06-1600
Skafjeld et al. (30)	Norway	RCT	41(21/20)	50	33/8	58.25	31.3	24.5	70	31.5	36.6	10.1186/s12902-015-0054-x
Petrova et al. (29)	UK	RCT	110(49/61)	48	82/26	62	31	NR	73	0	100	10.1111/dme.14152
Bus et al. (28)	Netherlands	RCT	304(151/153)	78	220/84	64.6	29.8	55.6	77	30.4	19.7	10.1136/bmjdrc-2021-002392
Qin et al. (27)	Indonesia	RCT	120(60/60)	24	58/62	60.7	24.18	18.3	95	18.3	33.3	10.1016/j.ijnurstu.2023.104571

BMI, body mass index; NR, not reported; PAD, peripheral arterial disease; RCT, randomized controlled trial; T2DM, type 2 diabetes mellitus.

**Figure 2:**
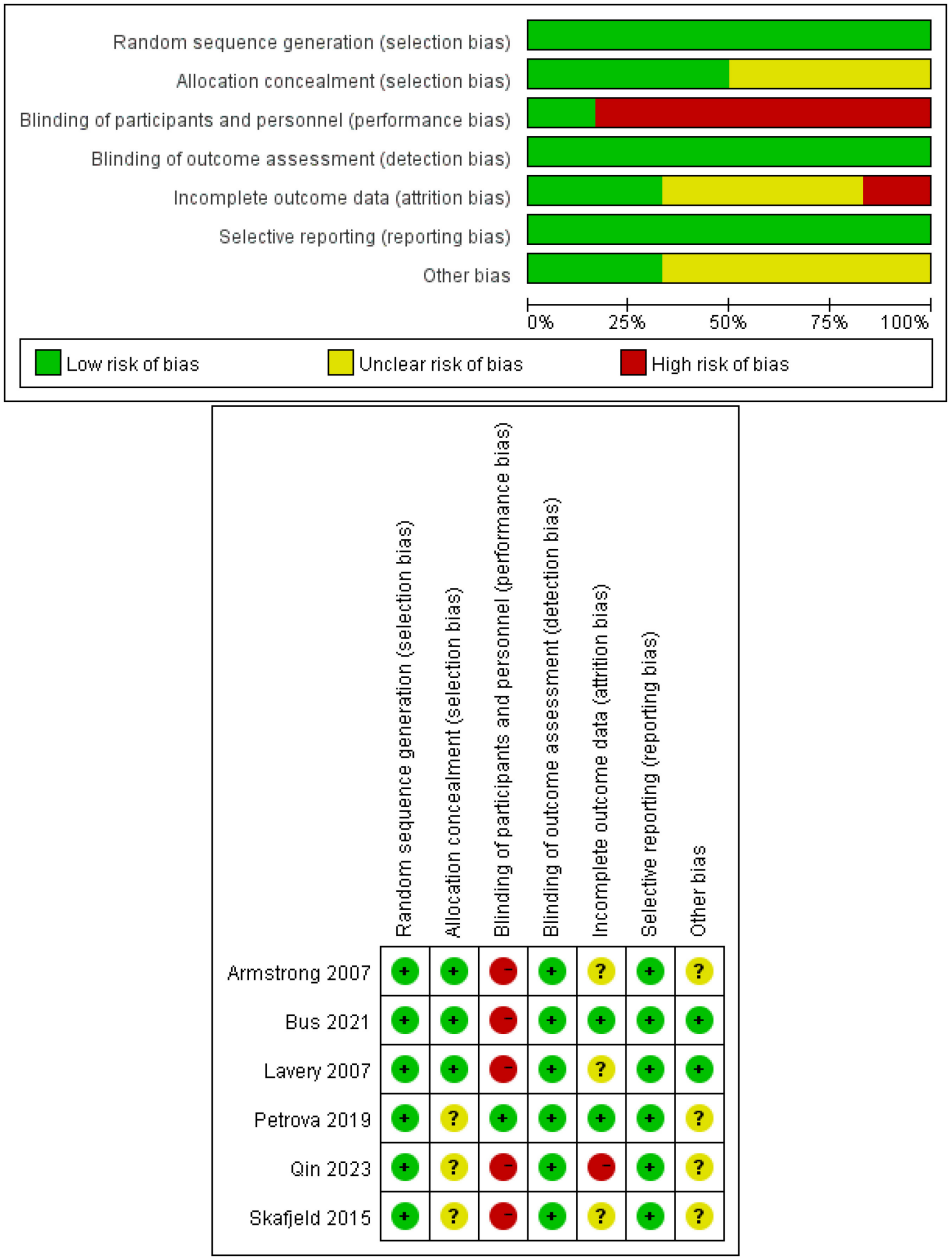
Quality assessment of included studies. Green, yellow, and red indicate low, moderate, and high risk of bias, respectively, in each domain.

The methodological quality of the six included trials was assessed using the Cochrane risk of bias tool. Overall, the quality of the evidence was considered acceptable. Most studies were judged to be at low risk of bias for random sequence generation and selective reporting. The risk of detection bias was also generally low, as outcome assessors were typically blinded to group allocation. The main potential source of bias was related to the blinding of participants and personnel, with the majority of studies rated as having a high risk of bias due to the behavioral nature of the temperature-guided preventive care intervention. A summary of the risk of bias assessment for each included study is provided in Figure 2.

### 3.2 Efficacy of temperature-guided preventive care for predefined outcomes

Time to the first foot ulcer recurrence (*I*^2^= 0%) and overall incidence of foot ulcer recurrence (*I*^2^= 41.44%) showed low heterogeneity; therefore, we used a fixed-effects model.

**Figure 3:**
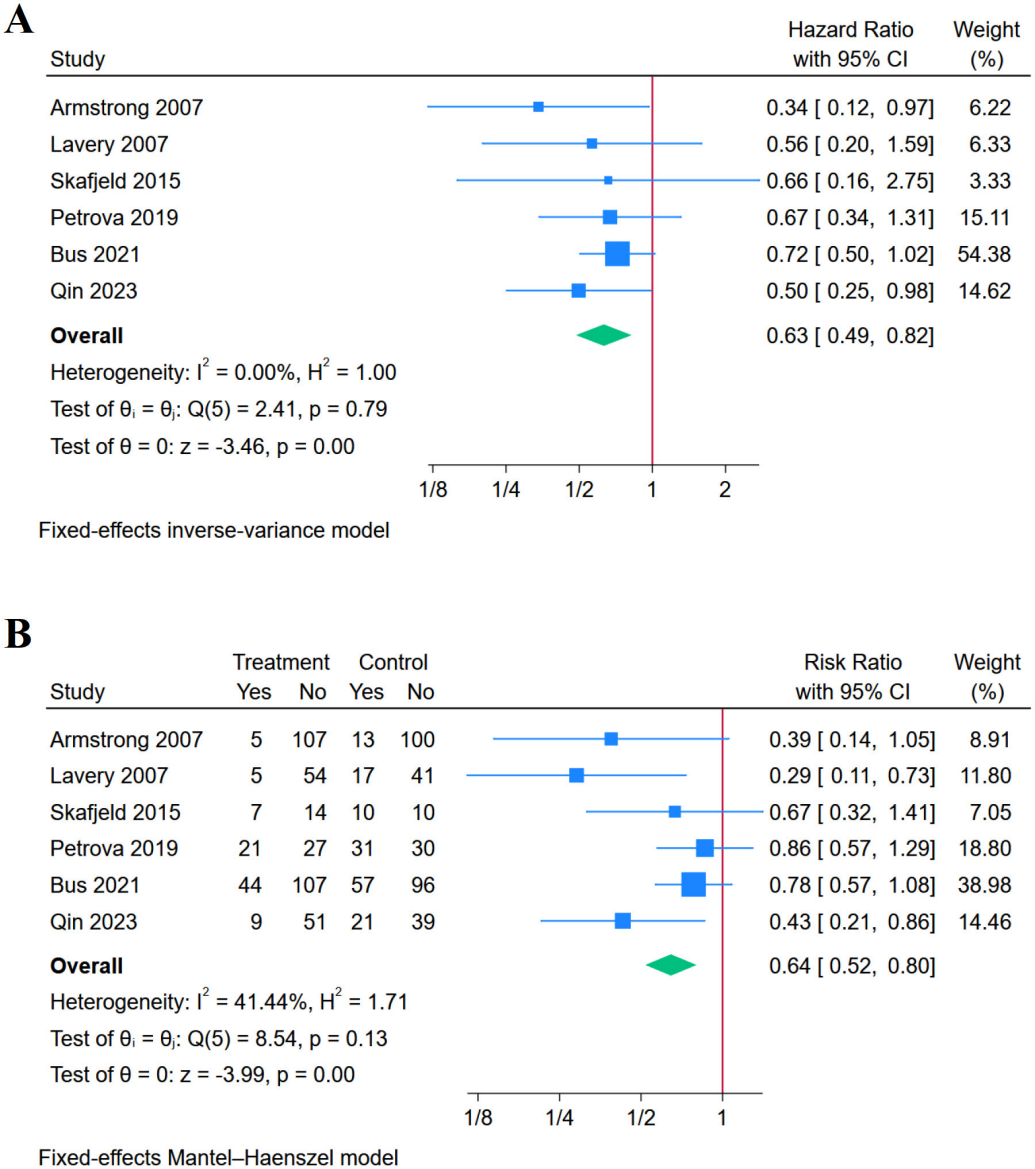
**(A)** Forest plot representing the HR for time to first foot ulcer recurrence and pooled HR with 95% Cl; **(B)** Forest plot representing the RR for overall incidence of foot ulcer recurrence and pooled RR with 95% Cl.

These 2 outcomes were reported in 6 studies involving 917 patients. The pooled analysis for the primary outcome, time to the first foot ulcer recurrence, demonstrated a statistically significant protective effect of temperature-guided preventive care. Participants in the intervention group had a significantly lower hazard of ulcer recurrence compared with those in the control group (HR 0.63; 95% CI: 0.49–0.82; *p* = 0.001). The forest plot for this analysis is presented in Figure 3A. For the secondary outcome, the overall incidence of foot ulcer recurrence, the risk of experiencing an ulcer recurrence was significantly lower in the intervention group than in the control group (RR 0.64; 95% CI: 0.52–0.80; *p* = 0.001). The forest plot for this outcome is shown in Figure 3B.

### 3.3 Sensitivity analysis and publication bias

To assess the stability of our findings, a leave-one-out sensitivity analysis was conducted. The direction and significance of the pooled effect estimate for both the primary and secondary outcomes remained consistent when each study was sequentially removed from the analysis, indicating that the results were not unduly influenced by any single study (Supplementary Figure 1). The potential for publication bias was evaluated through visual inspection of funnel plots and formally tested using Begg's regression test. The funnel plots for both outcomes appeared symmetrical (Supplementary Figure 2), and Begg's test did not indicate the presence of significant publication bias for either the primary (${\text{P}}_{\text{Beg}{\text{g}}^{\text{′}}\text{s}}$ = 0.26) or secondary outcome (${\text{P}}_{\text{Beg}{\text{g}}^{\text{′}}\text{s}}$ = 0.26).

## 4 Discussion

### 4.1 Principal findings

In this systematic review and meta-analysis of six randomized controlled trials involving 917 high-risk individuals with diabetes, temperature-guided preventive care based on skin temperature monitoring was associated with a significant reduction in both the time to first ulcer recurrence and the overall risk of recurrence compared with standard care. These findings support temperature-guided preventive care as an effective, patient-driven adjunct to current preventive strategies for recurrent DFU. As shown in Supplementary Table 1, abnormal temperature findings in the included trials generally triggered additional behavioral or clinical actions, such as activity reduction, contact with a study nurse or podiatrist, pressure-relief care, footwear advice, or individualized foot care. Therefore, the observed benefit should be interpreted as the effect of a temperature-guided preventive care strategy rather than that of temperature monitoring alone. From a clinical perspective, this benefit is biologically plausible, as monitoring local temperature may facilitate earlier recognition of pre-ulcerative inflammatory changes and allow timely intervention before overt skin breakdown occurs. Taken together, our results suggest that empowering patients with a simple and objective monitoring tool may help alter the clinical trajectory of this devastating complication.

### 4.2 Clinical and practical implications

Our meta-analysis included studies utilizing two different temperature-monitoring technologies: handheld infrared thermometers and thermal imagers. While both contributed to the overall positive effect in preventing DFU, the significant heterogeneity arising from different intervention protocols prevents a direct comparison of their efficacy. For instance, monitoring frequencies varied substantially, from daily self-monitoring in several studies (16, 18, 30) to monthly clinical assessment in another (29). This complexity makes it difficult to isolate the differential effects of the two measurement methods. However, from a clinical and economic standpoint, handheld thermometers represent a more accessible and cost-effective preventive measure, being significantly less expensive than thermal imagers. Their portability also facilitates daily patient use, aligning closely with the IWGDF guideline recommendations for at least daily foot inspection among individuals at moderate to high risk of ulceration. Our pooled analysis also confirmed that early detection of foot temperature changes significantly prolongs the time to ulcer recurrence. Although some of the included studies had smaller sample sizes (*n* < 100) (27, 29, 30), which resulted in notable differences in study weights and could potentially introduce bias, the survival curves of the intervention groups were consistently more favorable across all trials. This finding suggests a robust treatment effect. Future high-quality RCTs with larger sample sizes would be invaluable for increasing the precision of this estimate and reducing the heterogeneity among studies, thereby further strengthening the certainty and accuracy of the survival benefit conferred by this intervention.

However, despite this strong biological rationale, the clinical potential of temperature-guided preventive care has not yet been fully realized in routine practice. Current approaches still face challenges related to real-world deployability, standardization, and medical validation, and manual temperature measurements in particular may be difficult to interpret consistently outside research settings because of limited repeatability and reproducibility (14, 15). These issues should be considered when translating temperature-based monitoring from controlled or highly supervised settings into broader clinical and home-based preventive care settings.

Beyond efficacy, adherence is a key determinant of the real-world value of this patient-centered strategy. By providing a tool for timely and effective early warning, temperature-guided preventive care may help patients with DFU establish long-term monitoring habits, reduce their dependence on healthcare professionals and family members, and foster a sense of autonomy in long-term self-management, thereby improving adherence. Indeed, the literature suggests that adherence to this specific intervention is high, with studies reporting that 62.3% of users adhere to long-term temperature measurement and recording, and up to 87% adhere to daily monitoring (31, 32). While factors such as age, intervention duration, and the burden of self-management can influence adherence, it is noteworthy that older patients with DFU have been shown to exhibit higher adherence to self-management practices (33).

The rationale for temperature-guided preventive care is strongest in neuropathic diabetic foot disease, where repetitive mechanical stress on an insensate foot may cause subclinical inflammation and local temperature elevation before overt skin breakdown (7). However, local temperature changes may be more difficult to interpret in patients with predominant ischemic disease, peripheral artery disease (PAD), or chronic limb-threatening ischemia (CLTI), because skin temperature can be affected by impaired perfusion, infection, edema, revascularization status, and asymmetric or bilateral vascular disease (34). In our meta-analysis, PAD prevalence varied across studies, while patients with severe ischemia or CLTI were excluded in several trials and were generally underrepresented across the overall evidence base. Therefore, the current findings should be applied cautiously to patients with predominant ischemic or CLTI-related diabetic foot disease. Future studies should consider vascular status when defining temperature thresholds and developing preventive response protocols.

### 4.3 Limitations

However, our analysis is subject to several important limitations. First, the included studies varied significantly in their intervention protocols (e.g., daily self-monitoring vs. monthly clinical assessment), patient populations, and the quality and content of standard care provided to the control groups. This clinical and methodological heterogeneity is a potential source of bias and should be considered when interpreting the results. Second, while the individual trials were generally of good quality, the blinding of participants and personnel was not feasible by necessity, which introduces a potential for performance bias. Third, our meta-analysis is based on a limited number of studies. Therefore, the conclusions should be interpreted with caution, as the precision of the pooled results may be limited.

## 5 Conclusion

In conclusion, this systematic review and meta-analysis suggest that temperature-guided preventive care is an effective, patient-centered strategy for preventing DFU recurrence in high-risk individuals. It was associated with a reduced overall incidence of recurrence and a longer time to first ulcer recurrence. These findings support the potential value of using foot temperature-guided preventive care based on skin temperature monitoring as an early warning tool to guide timely preventive actions before overt ulceration develops.

Future research should further verify the accuracy, reliability, data transmission speed, compatibility with different products, clinical applicability, patient experience, and product price of emerging home foot temperature-monitoring technologies. When necessary, personalized customization can be developed to monitor patients' foot conditions, with the aim of early warning of the risk of diabetic foot ulcers or recurrence. This may provide more strategic options for high-risk patients with diabetic foot ulcers at home in the community to prevent the occurrence or recurrence of diabetic foot ulcers.

## Data Availability

The original contributions presented in the study are included in the article/Supplementary material, further inquiries can be directed to the corresponding author.

## Supplementary material

Supplementary Figure 1 Sensitivity analysis for evaluating the impact of individual studies on the overall heterogeneity and the pooled effect size, and assessing the stability of the results.

Supplementary Figure 2 Funnel plot for estimating the potential publication bias.

Supplementary Table 1 Temperature monitoring and intervention protocols of included studies.

## References

[1] GBD 2021 Diabetes Collaborators. Global, regional, and national burden of diabetes from 1990 to 2021, with projections of prevalence to 2050: a systematic analysis for the Global Burden of Disease Study 2021. Lancet. (2023) 402:203–34. doi: 10.1016/S0140-6736(23)01301-6 37356446 PMC10364581

[2] American Diabetes Association Professional Practice Committee. 12. Retinopathy, Neuropathy, and Foot Care: Standards of Care in Diabetes-2025. Diabetes Care. (2025) 48:S252–65. doi: 10.2337/dc25-S012 39651973 PMC11635040

[3] Cole JB Florez JC. Genetics of diabetes mellitus and diabetes complications. Nat Rev Nephrol. (2020) 16:377–90. doi: 10.1038/s41581-020-0278-5 32398868 PMC9639302

[4] Rinkel WD Luiten J van Dongen J Kuppens B Van Neck JW Polinder S . In-hospital costs of diabetic foot disease treated by a multidisciplinary foot team. Diabetes Res Clin Pract. (2017) 132:68–78. doi: 10.1016/j.diabres.2017.07.029 28802698

[5] Armstrong DG Tan TW Boulton AJM Bus SA. Diabetic foot ulcers: a review. Jama. (2023) 330:62–75. doi: 10.1001/jama.2023.10578 37395769 PMC10723802

[6] Armstrong DG Swerdlow MA Armstrong AA Conte MS Padula WV Bus SA. Five year mortality and direct costs of care for people with diabetic foot complications are comparable to cancer. J Foot Ankle Res. (2020) 13:16. doi: 10.1186/s13047-020-00383-2 32209136 PMC7092527

[7] Armstrong DG Boulton AJM Bus SA. Diabetic foot ulcers and their recurrence. N Engl J Med. (2017) 376:2367–75. doi: 10.1056/NEJMra1615439 28614678

[8] Vrátná E Husáková J Jarošíková R Dubský M Wosková V Bém R . Effects of a 12-week interventional exercise programme on muscle strength, mobility and fitness in patients with diabetic foot in remission: results from BIONEDIAN randomised controlled trial. Front Endocrinol. (2022) 13:869128. doi: 10.3389/fendo.2022.869128 35865313 PMC9294221

[9] Morbach S Furchert H Gröblinghoff U Hoffmeier H Kersten K Klauke GT . Long-term prognosis of diabetic foot patients and their limbs: amputation and death over the course of a decade. Diabetes Care. (2012) 35:2021–7. doi: 10.2337/dc12-0200 22815299 PMC3447849

[10] Armstrong DG Lavery LA Liswood PJ Todd WF Tredwell JA. Infrared dermal thermometry for the high-risk diabetic foot. Phys Ther. (1997) 77:169–75; discussion 76–7. doi: 10.1093/ptj/77.2.169 9037217

[11] Lazo-Porras M Bernabe-Ortiz A Sacksteder KA Gilman RH Malaga G Armstrong DG . Implementation of foot thermometry plus mHealth to prevent diabetic foot ulcers: study protocol for a randomized controlled trial. Trials. (2016) 17:206. doi: 10.1186/s13063-016-1333-1 27094007 PMC4837616

[12] Veneman T Schaper NC Bus SA. The concurrent validity, test-retest reliability and usability of a new foot temperature monitoring system for persons with diabetes at high risk of foot ulceration. Sensors. (2021) 21:3645. doi: 10.3390/s21113645 34073853 PMC8197257

[13] Anandhanarayanan A Teh K Goonoo M Tesfaye S Selvarajah D. Diabetic neuropathies. In:Feingold KR Adler RA Ahmed SF Anawalt B Blackman MR Chrousos G , editors. Endotext. South Dartmouth, MA: MDText.com, Inc. Copyright © 2000-2026, MDText.com, Inc. (2000).

[14] Lefaucheur JP. Screening and monitoring of diabetic polyneuropathy in clinical practice: present and future with connected devices. Front Neurol. (2025) 16:1679277. doi: 10.3389/fneur.2025.1679277 41195012 PMC12583224

[15] Jones PJ Lavery L Davies MJ Webb D Rowlands AV. Hotspots: adherence in home foot temperature monitoring interventions for at-risk feet with diabetes-A narrative review. Diabet Med. (2023) 40:e15189. doi: 10.1111/dme.15189 37489103

[16] Lavery LA Higgins KR Lanctot DR Constantinides GP Zamorano RG Athanasiou KA . Preventing diabetic foot ulcer recurrence in high-risk patients: use of temperature monitoring as a self-assessment tool. Diabetes Care. (2007) 30:14–20. doi: 10.2337/dc06-1600 17192326

[17] Reyzelman AM Koelewyn K Murphy M Shen X Yu E Pillai R . Continuous temperature-monitoring socks for home use in patients with diabetes: observational study. J Med Internet Res. (2018) 20:e12460. doi: 10.2196/12460 30559091 PMC6315272

[18] Armstrong DG Holtz-Neiderer K Wendel C Mohler MJ Kimbriel HR Lavery LA. Skin temperature monitoring reduces the risk for diabetic foot ulceration in high-risk patients. Am J Med. (2007) 120:1042–6. doi: 10.1016/j.amjmed.2007.06.028 18060924

[19] Lavery LA Higgins KR Lanctot DR Constantinides GP Zamorano RG Armstrong DG . Home monitoring of foot skin temperatures to prevent ulceration. Diabetes Care. (2004) 27:2642–7. doi: 10.2337/diacare.27.11.2642 15504999

[20] Aan de Stegge WB Mejaiti N van Netten JJ Dijkgraaf MGW van Baal JG Busch-Westbroek TE . The cost-effectiveness and cost-utility of at-home infrared temperature monitoring in reducing the incidence of foot ulcer recurrence in patients with diabetes (DIATEMP): study protocol for a randomized controlled trial. Trials. (2018) 19:520. doi: 10.1186/s13063-018-2890-2 30249296 PMC6154404

[21] Banks JL Petersen BJ Rothenberg GM Jong AS Page JC. Use of a remote temperature monitoring mat for the early identification of foot ulcers. Wounds. (2020) 32:44–9. 32155121

[22] Ming A Walter I Alhajjar A Leuckert M Mertens PR. Study protocol for a randomized controlled trial to test for preventive effects of diabetic foot ulceration by telemedicine that includes sensor-equipped insoles combined with photo documentation. Trials. (2019) 20:521. doi: 10.1186/s13063-019-3623-x 31439007 PMC6704693

[23] Najafi B Mohseni H Grewal GS Talal TK Menzies RA Armstrong DG. An optical-fiber-based smart textile (Smart Socks) to manage biomechanical risk factors associated with diabetic foot amputation. J Diabetes Sci Technol. (2017) 11:668–77. doi: 10.1177/1932296817709022 28513212 PMC5588846

[24] van Doremalen RFM van Netten JJ van Baal JG Vollenbroek-Hutten MMR van der Heijden F. Validation of low-cost smartphone-based thermal camera for diabetic foot assessment. Diabetes Res Clin Pract. (2019) 149:132–9. doi: 10.1016/j.diabres.2019.01.032 30738090

[25] Page MJ McKenzie JE Bossuyt PM Boutron I Hoffmann TC Mulrow CD . The PRISMA 2020 statement: an updated guideline for reporting systematic reviews. Bmj. (2021) 372:n71. doi: 10.1136/bmj.n71 33782057 PMC8005924

[26] Tierney JF Burdett S Fisher DJ. Practical methods for incorporating summary time-to-event data into meta-analysis: updated guidance. Syst Rev. (2025) 14:84. doi: 10.1186/s13643-025-02752-z 40211371 PMC11984287

[27] Qin Q Oe M Nakagami G Kashiwabara K Sugama J Sanada H . The effectiveness of a thermography-driven preventive foot care protocol on the recurrence of diabetic foot ulcers in low-medical resource settings: an open-labeled randomized controlled trial. Int J Nurs Stud. (2023) 146:104571. doi: 10.1016/j.ijnurstu.2023.104571 37586286

[28] Bus SA Aan de Stegge WB van Baal JG Busch-Westbroek TE Nollet F van Netten JJ. Effectiveness of at-home skin temperature monitoring in reducing the incidence of foot ulcer recurrence in people with diabetes: a multicenter randomized controlled trial (DIATEMP). BMJ Open Diabetes Res Care. (2021) 9:e002392. doi: 10.1136/bmjdrc-2021-002392 34493496 PMC8424833

[29] Petrova NL Donaldson NK Tang W MacDonald A Allen J Lomas C . Infrared thermography and ulcer prevention in the high-risk diabetic foot: data from a single-blind multicentre controlled clinical trial. Diabet Med. (2020) 37:95–104. doi: 10.1111/dme.14152 31629373

[30] Skafjeld A Iversen MM Holme I Ribu L Hvaal K Kilhovd BK . A pilot study testing the feasibility of skin temperature monitoring to reduce recurrent foot ulcers in patients with diabetes–a randomized controlled trial. BMC Endocr Disord. (2015) 15:55. doi: 10.1186/s12902-015-0054-x 26452544 PMC4600271

[31] Lazo-Porras M Bernabe-Ortiz A Taype-Rondan A Gilman RH Malaga G Manrique H . Foot thermometry with mHeath-based supplementation to prevent diabetic foot ulcers: a randomized controlled trial. Wellcome Open Res. (2020) 5:23. doi: 10.12688/wellcomeopenres.15531.1 32923686 PMC7463300

[32] Rovers FJ Van Netten JJ Busch-Westbroek TE Aan de Stegge WB Bus SA. Adherence to at-home monitoring of foot temperatures in people with diabetes at high risk of ulceration. Int J Low Extrem Wounds. (2025) 24:691–9. doi: 10.1177/15347346221114565 35840892 PMC12301524

[33] Waaijman R Keukenkamp R de Haart M Polomski WP Nollet F Bus SA. Adherence to wearing prescription custom-made footwear in patients with diabetes at high risk for plantar foot ulceration. Diabetes Care. (2013) 36:1613–8. doi: 10.2337/dc12-1330 23321218 PMC3661819

[34] Fitridge R Chuter V Mills J Hinchliffe R Azuma N Behrendt CA . The intersocietal IWGDF, ESVS, SVS guidelines on peripheral artery disease in people with diabetes mellitus and a foot ulcer. J Vasc Surg. (2023) 78:1101–31. doi: 10.1016/j.jvs.2023.07.020 37724985

