# Supplementary figures and images for: The efficacy of temperature-guided preventive care in reducing diabetic foot ulcer incidence and prolonging ulcer-free survival: a systematic review and meta-analysis of randomized controlled trials
Source: Front Med (Lausanne). 2026 Jun 22;13:1830535. doi: 10.3389/fmed.2026.1830535 (PMC13333436; doi:10.3389/fmed.2026.1830535)

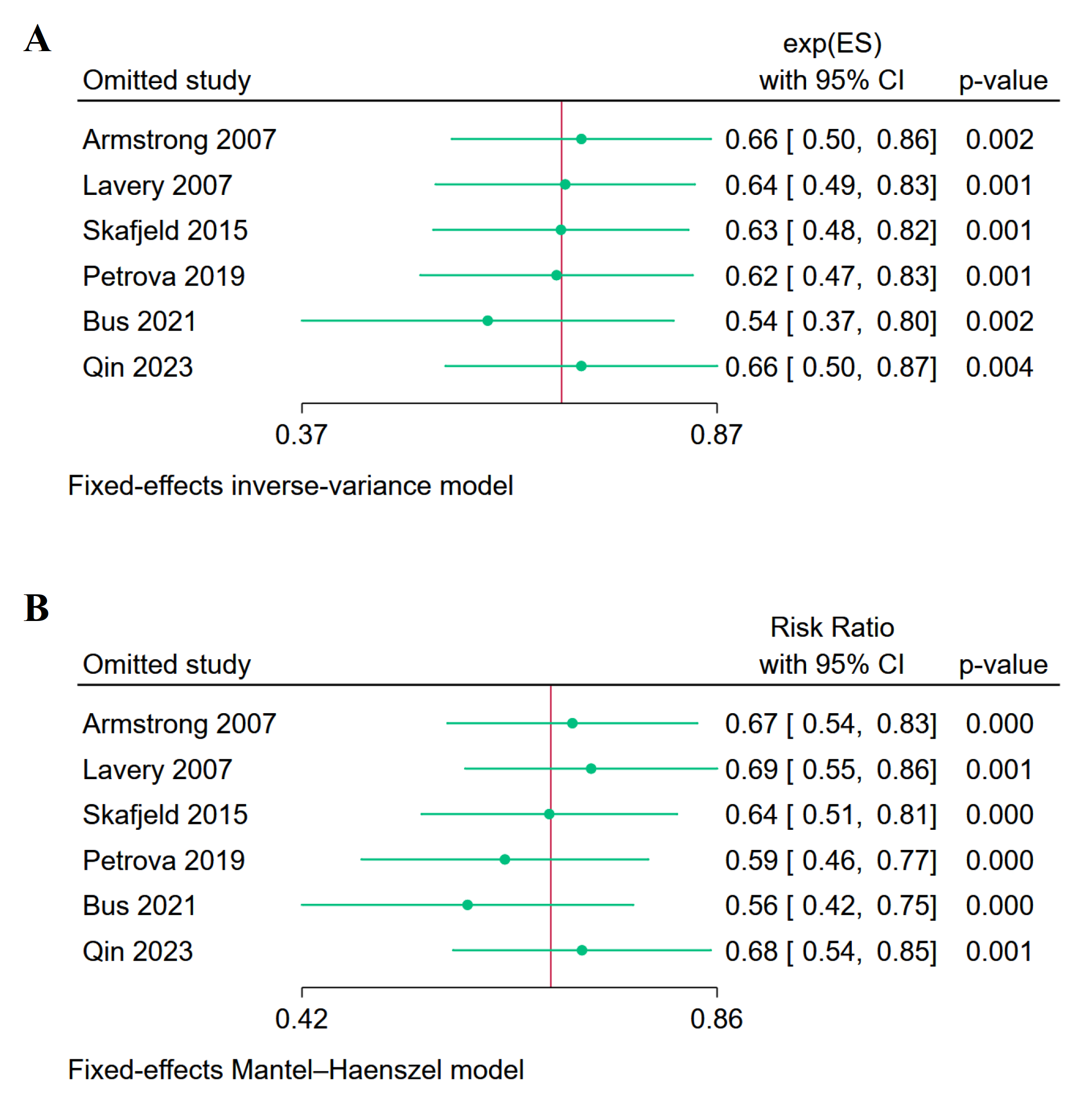

Supplement: Supplementary Figure 1 — Sensitivity analysis for evaluating the impact of individual studies on the overall heterogeneity and the pooled effect size, and assessing the stability of the results. [file Image_1.tif]

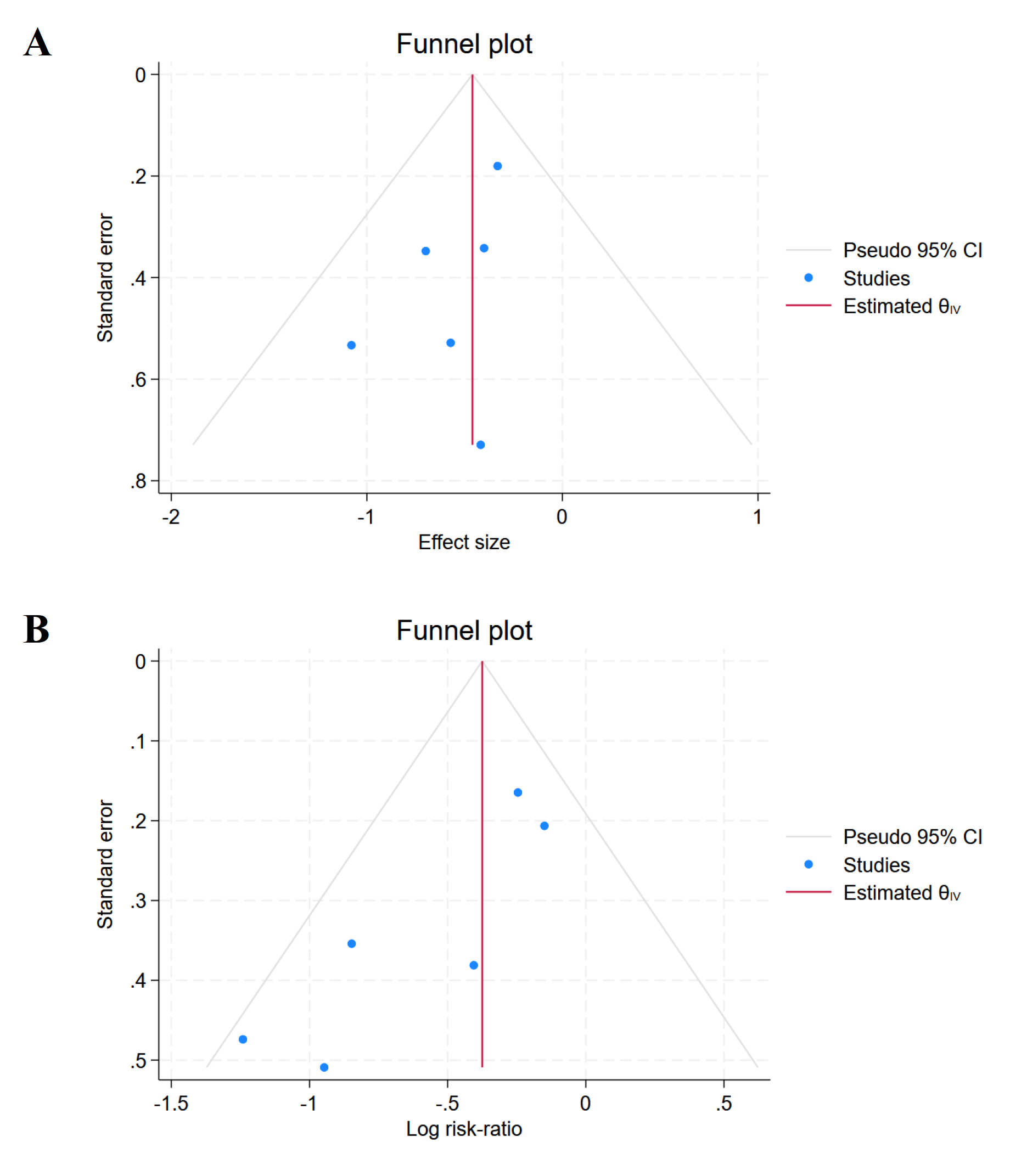

Supplement: Supplementary Figure 2 — Funnel plot for estimating the potential publication bias. [file Image_2.tif]
